# Supplementary figures and images for: Association of serum leptin and adiponectin concentrations with echocardiographic parameters and pathophysiological states in patients with cardiovascular disease receiving cardiovascular surgery
Source: PLoS One. 2019 Nov 8;14(11):e0225008. doi: 10.1371/journal.pone.0225008 (PMC6839852; doi:10.1371/journal.pone.0225008)

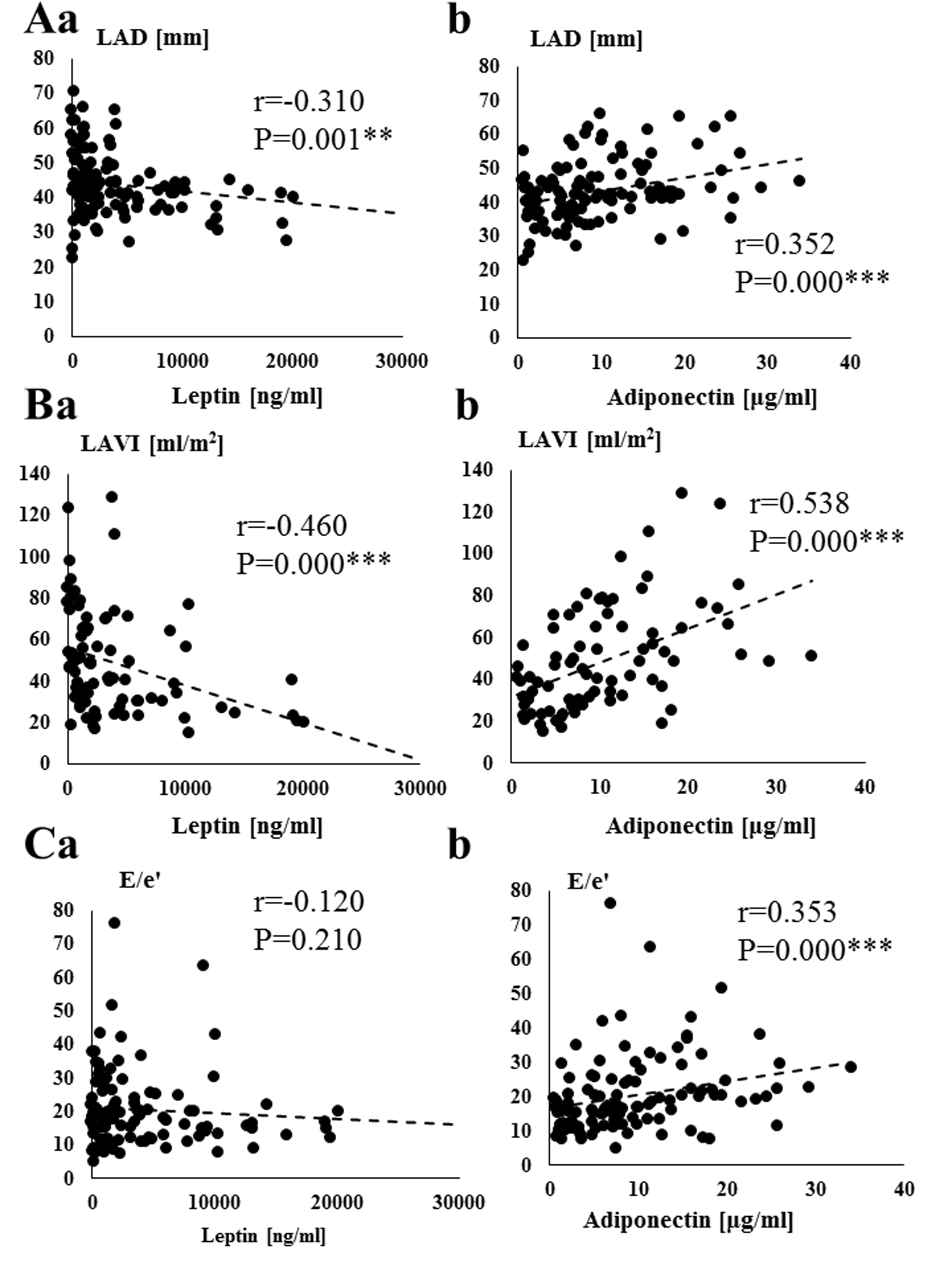

Supplement: S1 Fig — Relationships between the echocardiographic findings (LAD (A), LAVI (B), E/e’(C)) and serum leptin (Aa, Ba, Ca) and adiponectin levels (Ab, Bb, Cb) *P<0.05, **P<0.01, ***P<0.001. (TIF) [file pone.0225008.s001.TIF]
