# Supplementary material for: Association of serum leptin and adiponectin concentrations with echocardiographic parameters and pathophysiological states in patients with cardiovascular disease receiving cardiovascular surgery
Source: PLoS One. 2019 Nov 8;14(11):e0225008. doi: 10.1371/journal.pone.0225008 (PMC6839852; doi:10.1371/journal.pone.0225008)
Supplement: Attachment — Submitted filename: review-PLOS1-Adiponectin20190828.docx [file pone.0225008.s002.docx]

In this manuscript, the authors examined preoperative serum leptin and adiponectin levels, and their relationships to pathophysiological states, parameters of blood tests, and echocardiographic parameters in patients undergoing cardiac surgery. The authors demonstrated that multiple parameters and biomarkers that correlated with leptin and adiponectin concentrations. They also found that serum adiponectin concentrations were associated with sarcopenia, with a putative cut-off value of 4.94 mcg/ml. They concluded that leptin may be a cardioprotective adipokine that reduces cardiac remodeling without muscle wasting, whereas adiponectin plays in cardiac dysfunction and impaired metabolic signaling that us linked to heart failure progression.

First of all, I would like to congratulate the authors on their interesting paper, in which they extensively analyzed preoperative leptin and adiponectin. However, it raises several important concerns that need to be addressed:

1. Cardiac functions and echocardiographic findings are strongly influenced by conditions and the severity of cardiac diseases. As the authors stated in limitation, the patients’ underlying backgrounds, general and cardiac conditions and medications vary, and their adiponectin and leptin levels were determined only preoperatively. Therefore, it is unclear whether leptin and adiponectin play causative roles in CVD. In other words, the authors should be careful of leading conclusions that these parameters are “cardioprotective” or “cardiodepressive” in their patient series. For example, BNP is the most reliable biomarker for cardiac strain and the severity of heart failure, and it is known to work as a potential cardioprotective peptide. The authors should carefully interpret the data, and explain why they concluded leptin might be “cardioprotective” and reduces cardiac remodeling, as opposed to previous reports as well as their background. Readers would be more interested in whether leptin and adiponectin work as potential biomarkers of the severity of cardiac diseases, and how they change after surgery.
2. They also presented the relationship between the adipokines and patients’ physical status and sarcopenia. This is a different story from cardiac functions but I personally find this part rather interesting. Since there are many questions raised in this context, I would suggest the authors present the cardiac part and sarcopenia in different papers. Otherwise, the authors may present a diagram showing putative interactions between cardiac diseases, cardioprotections and sarcopenia in Discussion. Since age is a cofounder of both adiponectin (in their data) and sarcopenia, is it possible a positive correlation of adiponectin with sarcopenia is due to age? Were there any relationship between gender and the prevalence of sarcopenia? The authors should clarify whether the cut-off value is applied regardless of age?　Is it applied to both male and female?
3. Some data they presented were redundant, which may have made the paper much more complicating. For example, both Figure 1 and 2 were duplicates of Table 3. I am not sure if it is necessary to present Model1-3 in Table 4. The authors presented relationships between serum adiponectin levels and GDF-15, TNF-alpha, d-ROMs. I would not find the data necessary, unless the authors examine potential roles of the relatively unfamiliar markers in their patient series.

Minor point: TNPα(p11, line 15) is misspelled.
